# Understanding the Progression of Chronic Kidney Disease in Cats: From Pathophysiology to Emerging Biomarkers

**DOI:** 10.3390/vetsci13020199

**Published:** 2026-02-19

**Authors:** Sofia Rosa, Ana C. Silvestre-Ferreira, Rui Martins, Felisbina Pereira Queiroga

**Affiliations:** 1 Animal and Veterinary Science Research Centre (CECAV), University of Trás-os-Montes and Alto Douro (UTAD), 5001-801 Vila Real, Portugal; sofiaasrosa99@gmail.com (S.R.); aferreir@utad.pt (A.C.S.-F.); 2 Department of Veterinary Sciences, University of Trás-os-Montes and Alto Douro (UTAD), 5001-801 Vila Real, Portugal; 3 Associate Laboratory for Animal and Veterinary Sciences (AL4AnimalS), 1300-477 Lisboa, Portugal; 4 Institute for Systems and Computer Engineering, Technology and Science (INESC TEC), 4200-465 Porto, Portugal; rmcm@inesctec.pt; 5 Center for the Study of Animal Sciences (CECA-ICETA), University of Porto, 4099-002 Porto, Portugal

**Keywords:** feline chronic kidney disease, renal fibrosis, biomarkers, SDMA, FGF-23, International Renal Interest Society staging, cystatin B, geriatric cats

## Abstract

Feline chronic kidney disease is a leading cause of mortality in geriatric cats, characterized by a progressive and irreversible loss of renal function. Despite its high prevalence, early diagnosis remains challenging due to nephron compensatory mechanisms and the limited sensitivity of traditional biomarkers, creating a diagnostic gap that necessitates the exploration of novel biomarkers for earlier detection. This review examines the complex pathophysiology of the disease, including renin–angiotensin–aldosterone system activation, tubulointerstitial fibrosis, and mineral metabolism disturbances. By analyzing recent scientific literature, this work evaluates current diagnostic landscape and clinical relevance of emerging biomarkers. Evidence indicates that symmetric dimethylarginine and fibroblast growth factor-23 improve detection of early metabolic and filtration changes, while urinary biomarkers like cystatin B and retinol-binding protein provide specific insights into tubular injury. Bridging the diagnostic gap requires a transition from a reactive, azotemia-based framework to a multi-parametric diagnostic approach that integrates novel biomarkers with serial clinical and laboratory monitoring. Although financial constraints and limited availability restrict widespread clinical implementation, incorporating these advances is essential for earlier prognostic stratification and timely therapeutic decision-making. This integrated strategy has the potential to slow disease progression and improve survival and quality of life in cats with chronic kidney disease.

## Simple Summary

Chronic kidney disease is one of the most common medical conditions in older cats, leading to a gradual and permanent loss of kidney function. Unfortunately, by the time a cat shows signs of illness or traditional blood tests detect a problem, a large portion of the kidneys may already be damaged. This review focuses on the mechanisms underlying disease progression and critically examines emerging biomarkers with the potential to detect renal dysfunction at earlier stages than currently available methods. By reviewing the latest scientific research, we highlight how these tools can help veterinarians detect the disease in its silent stages. Earlier identification allows not only earlier intervention but also more accurate monitoring of disease progression. This shift enables veterinarians to move from a reactive approach toward proactive management strategies, which may significantly extend survival time and improve the quality of life of aging feline patients.

## 1. Introduction

### 1.1. Feline Chronic Kidney Disease (CKD): Epidemiology, Etiology and Risk Factors

Chronic kidney disease (CKD) is a heterogeneous disease syndrome defined by progressive renal damage that encompasses a wide range of renal pathologies and etiologies [1,2]. It is characterized by the presence of either structural or functional abnormalities in one or both kidneys, which have been present for a prolonged period, generally lasting three months or more. It can occur with a range of severity levels, from minor structural lesions in a single kidney to significant nephron loss in both kidneys [3]. Despite extensive characterization, the initiating cause of feline CKD often remains unclear, and the disease is best understood as a multifactorial and progressive continuum rather than a single pathological entity.

This disease is one of the most common in cats and the number of cases has increased in recent years due to better awareness and developments in diagnostic methods [4,5,6]. Nevertheless, this apparent increase likely reflects improved recognition rather than a true rise in incidence, particularly in aging feline populations.

Data on the exact prevalence of CKD in cats are still scarce [4,5]. Studies show that CKD affects between 1% and 3% of cats in general veterinary practices, but this number can reach up to 50% depending on the population and diagnostic criteria used [4]. The prevalence increases with age, with the highest rates in cats over 15 years old, ranging from 31% to 80% depending on the study [5,7]. In the UK, kidney disease was the most common cause of death in cats older than 5 years, making up 12.1% of cases [1,4]. Notably, CKD is twice as prevalent in geriatric cats as in age-matched canine populations [4], highlighting important species-specific susceptibilities that remain incompletely understood.

The etiology of feline CKD is frequently idiopathic, despite the identification of multiple factors associated with an increased risk of disease development [4]. One of those is breed predisposition, with purebred cats showing a greater susceptibility than non-pedigree cats, such as Domestic Shorthairs [8,9]. Certain breeds, such as the Birman, tend to have higher serum creatinine concentrations, which could lead to misclassification in these studies [10]. The most prevalent breeds affected by CKD include Maine Coon, Abyssinian, Siamese, Russian Blue, and Burmese cats [9,11]. In addition, specific genetic mutations can also contribute to the development of CKD, such as the mutation in the polycystic kidney disease 1 (PKD1) gene, which is highly prevalent in Persian (46%), Scottish Fold (54%), and American Shorthair cats (47%) [12,13]. However, the presence of genetic risk does not fully explain disease onset or progression, reinforcing the multifactorial nature of feline CKD.

Age represents a further critical risk factor for feline CKD. The disease is particularly common in geriatric cats, with the prevalence starting to increase from the age of 5 to 6 years [4,5]. In young cats, CKD development is primarily attributed to hereditary diseases, while in geriatric felines, it is often driven by comorbidities that impair renal function [14]. Demographic variables, such as sex, also modulate CKD risk. Male cats are more susceptible to developing urethral obstruction than females, which can result in kidney injury and subsequently increase the risk of CKD. However, studies have shown that the likelihood of being diagnosed with CKD does not significantly differ between neutered and intact males. Nevertheless, when comparing sexes, neutered males are more likely to be diagnosed with CKD compared to spayed females, suggesting that sex-related hormonal or behavioral factors may influence disease susceptibility rather than acting as direct causal determinants [8,9,14].

Diet and body condition are also important factors when it comes to feline CKD risk [15]. Cats with a thin body condition or those that are frequently dehydrated tend to have a higher probability of developing the disease [8]. Various studies have investigated how diet influences this risk; for example, feeding cats exclusively commercial dry food has been associated with an increased risk of CKD [11,15]. On the other hand, factors such as free feeding, low fiber intake, high protein, and low potassium levels may increase the risk of developing CKD [10]. These associations, however, remain controversial and may be influenced by differences in study design and dietary recall bias. This is particularly relevant as owners of cats already diagnosed with CKD may retrospectively over-emphasize previous dietary choices, complicating firm causal conclusions.

Specific interventions, such as the administration of antibiotics and non-steroidal anti-inflammatory drugs (NSAIDs) including those used for dental disease, can directly contribute to renal impairment. Similarly, the general anesthesia required for these procedures carries an inherent risk of kidney injury, with both factors collectively increasing the likelihood of developing CKD [8,10]. Regarding preventive medicine, the potential association between vaccination and feline CKD remains a subject of ongoing debate. Rather than a direct causal link to the vaccine product itself, the issue likely reflects a cumulative inflammatory burden on the renal parenchyma. As a highly vascularized organ, the feline kidney is particularly susceptible to immune complex deposition and secondary interstitial inflammation, often acting as a ‘bystander victim’ of systemic immune stimulation. Consequently, further research into pathophysiological mechanisms, such as renal-specific antibodies, is required to determine if adjusting vaccination protocols by balancing essential protection with the need to minimize unnecessary systemic inflammation can effectively preserve renal function [10].

Beyond demographic and lifestyle-related factors, a variety of underlying diseases and pathological conditions have also been implicated in the development of CKD in cats. Both congenital forms of CKD, including hereditary conditions such as polycystic kidney disease and renal dysplasia, and acquired forms, resulting from infections, toxic insults, or chronic inflammation, contribute to the complex etiology of the disease in feline populations. In this context, a broad range of primary renal disorders, such as amyloidosis, juvenile renal dysplasia, glomerular diseases, polycystic kidney disease, nephro- and ureterolithiasis, and lymphoma, have been identified as potential contributors. Chronic dietary imbalances and bacterial pyelonephritis are likewise recognized as relevant etiologies [4,16].

Other systemic conditions are also frequently seen in cats with CKD and may contribute to its development. Systemic hypertension, cardiovascular disease, and primary hyperaldosteronism are among the most commonly reported [16,17,18]. High blood pressure is found in approximately 20–65% of cats with CKD and is often linked to proteinuria, though it does not seem to predict survival or cause major kidney damage in treated cats [16,19]. Heart disease, such as hypertrophic cardiomyopathy, is also frequent: around 46.6% of cats with CKD have left ventricular hypertrophy, 59% of cats with hypertrophic cardiomyopathy are azotemic, and 12.7% have CKD. Systolic arterial pressure is also higher in azotemic than in non-azotemic cats with this condition. In addition, primary hyperaldosteronism can lead to mild azotemia, hypokalemia, systemic hypertension, and progressive kidney damage, with histological changes similar to those observed in humans. Although these conditions frequently coexist with CKD, their role as primary risk factors versus parallel comorbidities remains incompletely defined [16,20].

Infectious agents may further contribute to the pathogenesis of CKD. Several viral infections, including feline immunodeficiency virus (FIV), feline leukemia virus (FeLV), feline infectious peritonitis virus (FIP), feline morbillivirus (FeMV), and foamy virus (FFV), have all been associated with renal damage and may promote the progression of chronic kidney injury [4,5,14]. Bacterial infections, such as leptospirosis and bartonellosis, as well as parasitic diseases like leishmaniosis and heartworm disease, have also been implicated [5,14].

Urinary tract infections, including bacterial pyelonephritis, may also play a contributory role in renal deterioration [4,14]. Another condition increasingly recognized for its potential systemic impact is periodontal disease (PD). Retrospective studies have shown that cats with advanced PD (especially those in stages 3 or 4) have a significantly higher risk of developing CKD [9,10].

### 1.2. Pathophysiology of Feline Chronic Kidney Disease Progression

**Figure 1:**
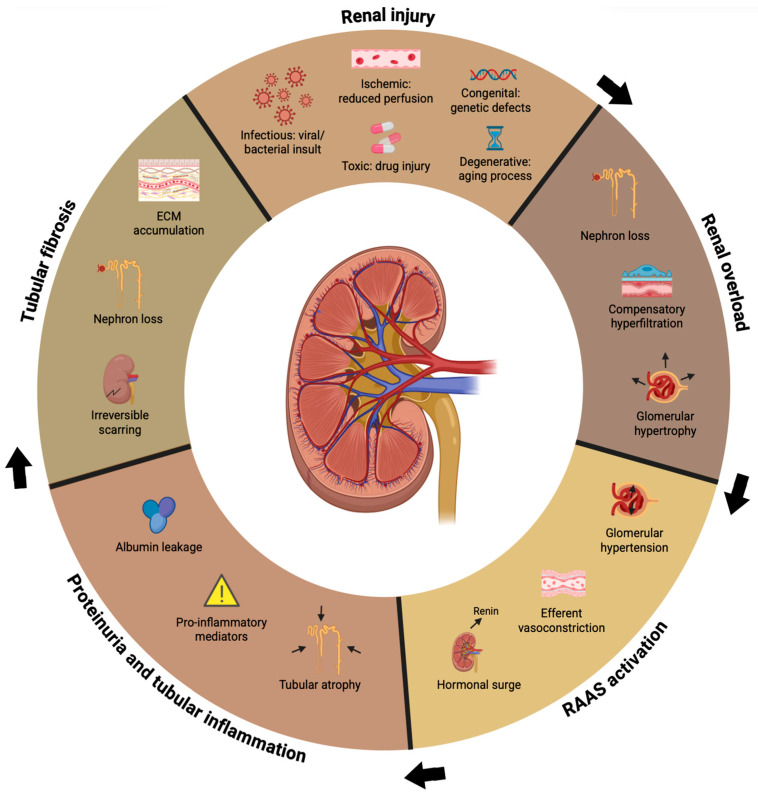
Self-perpetuating cycle of feline CKD progression. The arrows indicate a clockwise sequence, starting with the initial ‘Renal injury’ (top) and progressing through compensatory and inflammatory stages until reaching ‘Tubular fibrosis’ (left). Initial renal injury triggers compensatory mechanisms, including increased single-nephron glomerular filtration rate (GFR), glomerular hypertension, and activation of the renin–angiotensin–aldosterone system (RAAS). Although initially adaptive, sustained activation of these pathways promotes proteinuria, chronic inflammation, tissue hypoxia, and oxidative stress, ultimately leading to progressive tubulointerstitial fibrosis and irreversible nephron loss. These interconnected processes establish a vicious cycle that drives CKD progression regardless of the initiating renal insult. This fibrosis and loss of functional units represent the final stage of the cycle, which impairs the remaining nephrons and perpetuates the clinical state of kidney failure. ECM, extracellular matrix.

CKD in cats progresses through a self-perpetuating cycle of structural and functional alterations in the residual nephrons after injury (Figure 1). Compensatory tools such as increased single-nephron glomerular filtration rate (GFR), glomerular hypertension, and hypertrophy help delay the start of azotemia until more than 75% of renal function is lost. While these adaptive responses are essential for short-term homeostasis, they ultimately become maladaptive, contributing to progressive nephron injury and accelerating disease progression [16].

As GFR decreases, uremic toxins accumulate, worsening clinical signs and directly promoting further renal injury [16]. Feline CKD is therefore best conceptualized as a dynamic process driven by the interplay between hemodynamic stress, chronic inflammation, and progressive fibrotic remodeling, rather than a static condition defined solely by biochemical thresholds. This pathophysiological cascade is largely independent of the initiating cause and represents a common final pathway shared across diverse renal insults [4,14,16]. 

Renal fibrosis plays a significant role in the progression of CKD and represents the principal irreversible lesion responsible for functional decline. In healthy kidneys, the interstitium contains fibroblasts and dendritic cells within an extracellular matrix (ECM) rich in collagens (I, III, VII), fibronectin, and glycoproteins such as tenascin. Following renal injury, inflammatory signaling triggers fibroblast activation, ECM expansion, and tissue scarring. Although fibrosis may initially provide structural support during attempted tissue repair, its persistence and progression ultimately disrupt normal renal architecture and function. Histologically, this process is characterized by excessive ECM accumulation, loss of peritubular capillaries, chronic inflammation, tubular atrophy and dilation, and, in advanced stages, mineralization [14,21]. Myofibroblasts, derived from resident fibroblasts, pericytes, or through epithelial–mesenchymal transition, are key effector cells in renal fibrogenesis. These cells synthesize large amounts of ECM proteins, including collagen IV and fibronectin, and produce profibrotic mediators such as transforming growth factor-beta (TGF-β) [14]. Although the feline kidney retains a limited regenerative capacity, repetitive or severe injury, such as ischemic events or bacterial pyelonephritis, overwhelms reparative mechanisms and results in permanent scarring [21].

Proteinuria further amplifies this fibrotic cascade. Increased glomerular permeability leads to an overload of the megalin and cubulin-mediated endocytosis system, causing tubular epithelial cells to transition from an adaptive response to a pro-inflammatory state. This triggers the release of cytokines and the recruitment of inflammatory cells, ultimately promoting collagen deposition and progressive renal loss [20]. This maladaptive tubular response links glomerular injury to tubulointerstitial inflammation, reinforcing fibrosis even in a species where primary glomerular disease is relatively uncommon. The transition toward a profibrotic phenotype is largely driven by transforming growth factor-beta 1 (TGF-β1), a master regulator of ECM synthesis and degradation. TGF-β1 expression is upregulated by multiple stimuli, including renin–angiotensin–aldosterone system (RAAS) activation, oxidative stress, hypoxia, and sustained proteinuria. In cats, urinary TGF-β1 normalized to creatinine (TGF-β1:UCrR) has been associated with higher serum creatinine concentrations. However, its clinical utility remains limited due to the lack of direct histopathological correlation and standardized reference intervals, restricting its use primarily to research settings [22]. Another mediator increasingly implicated in renal fibrosis is transglutaminase-2 (TG-2), an enzyme that stabilizes the ECM by cross-linking its protein components, thereby enhancing resistance to proteolytic degradation. TG-2 also facilitates the activation of latent TGF-β1, further reinforcing fibrotic signaling pathways. In cats with CKD, increased TG-2 activity has been correlated with elevated serum creatinine, urea, and phosphorus concentrations, suggesting a contributory role in disease severity and highlighting TG-2 as a potential future therapeutic target [21].

#### 1.2.1. Kidney Morphologic Changes

In geriatric cats with CKD, histological lesions are predominantly localized within the tubulo-interstitial compartment, with glomerular sclerotic changes appearing mostly as secondary manifestations. Histopathologically, these lesions are multifocal to segmental and include infiltration of mononuclear cells in the interstitium, tubular degeneration and atrophy, interstitial fibrosis, mineralization of Bowman’s capsule and tubular basement membranes, and accumulation of lipids in the interstitium and glomerulosclerosis.

Tubular atrophy, interstitial inflammation, and fibrosis are consistent findings that progressively worsen alongside disease progression. Atrophic cortical tubules typically appear in clusters, exhibiting basement membrane alterations such as thickening and wrinkling or, conversely, thinning. Microscopically, these atrophic tubules are characterized by a marked reduction in epithelial height and luminal dilation. The interstitial space becomes expanded by pale eosinophilic collagen fibers, indicating fibrosis, which displaces the normal parenchyma. This area is often infiltrated by multifocal clusters of mononuclear cells, primarily lymphocytes and plasma cells, signifying chronic active inflammation [4]. Interstitial lipid accumulation, frequently observed in IRIS (International Renal Interest Society) stages 2 to 4, is often associated with granulomatous inflammation; this is likely a consequence of tubular ischemia and subsequent rupture, leading to the release of intraepithelial lipids [23]. Notably, the extent of interstitial fibrosis correlates closely with the severity of azotemia, becoming most prominent in IRIS stage 4 [4].

From a glomerular perspective, affected kidneys often demonstrate compensatory hypertrophy and expansion of the mesangial matrix. While significant nephron loss drives hyperperfusion and potential podocyte damage consistent with focal segmental glomerulosclerosis (FSGS), this secondary manifestation is relatively mild in the feline species. In spontaneous CKD, FSGS typically affects only a limited proportion of glomeruli and capillary tufts. More commonly, glomeruli undergo global sclerosis or obsolescence. Although global sclerosis is a feature of normal aging, its severity is markedly exacerbated in CKD. These sclerotic glomeruli exhibit collapse of the capillary tuft and fibrosis of the urinary space, findings which are more consistent with ischemic glomerular obsolescence than with classic FSGS progression. In advanced stages, these shrunken glomeruli may become indistinguishable from the surrounding fibrotic stroma [4,24].

Structural vascular alterations further complicate the disease profile. Cats with reduced renal mass often exhibit preferential dilation of afferent arterioles, contributing to glomerular hypertension and hyperfiltration in functional nephrons [4]. Furthermore, hypertensive cats frequently display vascular lesions such as hyaline and hyperplastic arteriosclerosis [24]. Ultimately, these structural changes are driven by the activation of myofibroblasts, which can be derived from pericytes, fibroblasts, or via epithelial–mesenchymal transition. These cells deposit substantial quantities of extracellular matrix components, such as collagen and fibronectin, which directly correlate with clinical renal dysfunction [16].

#### 1.2.2. RAAS Activation, Hypoxia, and Oxidative Stress

**Figure 2:**
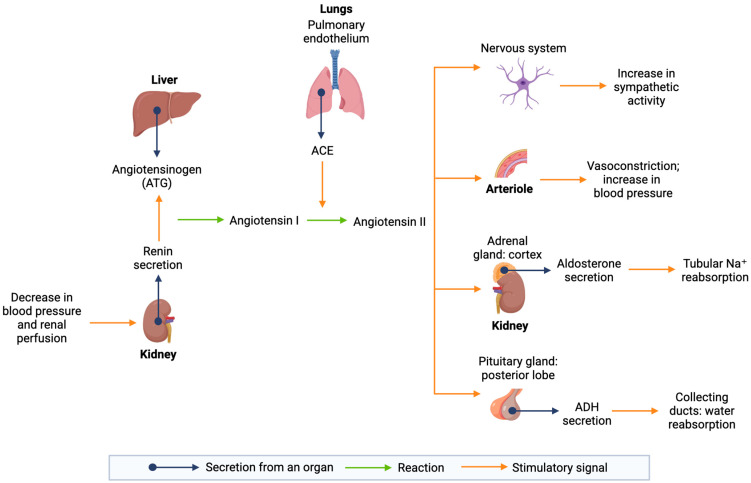
Schematic representation of the renin–angiotensin–aldosterone system (RAAS) and its main physiological effects. Renin is released from the juxtaglomerular cells of the kidney in response to decreased renal perfusion pressure, reduced NaCl delivery to the macula densa, and increased sympathetic stimulation. Renin catalyzes the conversion of angiotensinogen, synthesized by the liver, into angiotensin I. Angiotensin I is subsequently converted into angiotensin II by angiotensin-converting enzyme (ACE), primarily expressed in the pulmonary capillary endothelium. Angiotensin II exerts multiple physiological effects, including arteriolar vasoconstriction with increased blood pressure, stimulation of aldosterone secretion from the adrenal cortex leading to increased sodium reabsorption and potassium excretion in the distal tubules and collecting ducts, stimulation of antidiuretic hormone (ADH) release from the posterior pituitary with enhanced water reabsorption in the collecting ducts, and increased sympathetic activity. This schematic representation summarizes the major components of the RAAS and does not depict all intermediate pathways or feedback mechanisms.

The RAAS (Figure 2) is a fundamental endocrine mechanism that regulates blood pressure, sodium levels, and extracellular fluid balance [25]. Under physiological conditions, it ensures stable GFR and renal perfusion. When blood pressure or renal perfusion falls, juxtaglomerular cells release renin, an enzyme that catalyzes the conversion of angiotensinogen (ATG) into angiotensin I. This precursor is subsequently converted into angiotensin II by the angiotensin-converting enzyme (ACE), which is highly active in the endothelial cells of the lungs and within the proximal renal tubules [26,27].

Angiotensin II acts as a potent vasoconstrictor and plays a central role in renal hemodynamics through its interaction with the angiotensin type 1 (AT1) receptor. By inducing vasoconstriction of the efferent arteriole, it increases glomerular capillary pressure to maintain a stable GFR even during systemic hypotension. Furthermore, angiotensin II stimulates aldosterone secretion, promoting sodium and water reabsorption, and triggers the release of antidiuretic hormone (ADH) to enhance fluid retention. While these mechanisms are essential for homeostasis, their chronic activation in feline CKD becomes a primary driver of disease progression [20,26,28].

In cats with CKD, RAAS activation is a prominent feature both systemically and intrarenally. The feline kidney possesses all components necessary for local activation, and intrarenal angiotensin II levels often significantly exceed plasma concentrations, making systemic measurements insufficient to assess the true renal status [14,21,29]. Although initially compensatory, this response becomes maladaptive, contributing to glomerular hypertension, hyperfiltration, and proteinuria [29]. Other vasoconstrictors, such as thromboxane A2 and endothelin-1 (ET-1), may exacerbate these effects and promote segmental tubular hypoxia, particularly in geriatric cats [4,29].

Beyond its hemodynamic effects, angiotensin II also acts as a potent pro-fibrotic mediator, driving chronic structural damage independently of its effects on blood pressure. By triggering the activation of key growth factors, it promotes the transformation of renal cells into fibroblasts and the progressive accumulation of scar tissue [21]. Additionally, endothelial dysfunction impairs the release of nitric oxide (NO), compromising renal microcirculation and reducing renal oxygenation [17]. Notably, calcitriol serves as a negative regulator of the RAAS by suppressing renin transcription and reducing angiotensinogen expression. Through vitamin D receptor activation (VDRA), it limits fibrosis and inflammation by inhibiting NF-κB and restoring Klotho expression, which is typically downregulated by angiotensin II [29].

These hemodynamic alterations directly contribute to renal hypoxia, a critical disorder in feline CKD progression. Despite receiving a large blood supply, the renal medulla naturally operates at low oxygen levels. As the disease advances, glomerulosclerosis and RAAS-mediated efferent vasoconstriction disrupt blood flow to the peritubular capillaries, further reducing oxygen delivery to the renal tissue. In response, kidney cells adapt by promoting fibrosis and the epithelial-to-mesenchymal transition (EMT), creating a vicious cycle where worsening hypoxia accelerates tissue damage, a state often compounded by concurrent anemia [14].

Closely linked to hypoxia is oxidative stress, which arises from an imbalance between the production of reactive oxygen species (ROS) and the capacity of antioxidant defense systems. The feline kidney is particularly vulnerable due to its high metabolic rate; in CKD, this susceptibility is exacerbated by compensatory hyperfunction of surviving nephrons, leading to increased cellular oxygen consumption and ROS production. This process is further stimulated by angiotensin II and chronic inflammation. Excess ROS, including superoxide anions and hydrogen peroxide, damage DNA, lipids, and proteins, ultimately inducing apoptosis, necrosis, and progressive fibrotic remodeling [14,30].

Evidence of oxidative stress in feline CKD includes increased GSH:GSSG ratios and elevated glutathione peroxidase activity in advanced IRIS stages [31,32]. Furthermore, proteinuria contributes to oxidative stress, as reabsorbed proteins stimulate tubular cells to produce pro-inflammatory cytokines. Although clinical studies evaluating antioxidant supplementation in cats remain limited, dietary supplementation with vitamins C and E in elderly cats has shown a significant reduction in DNA damage, highlighting the potential role of antioxidant strategies in mitigating CKD progression [14,33].

#### 1.2.3. Proteinuria and Mineral Disorders

Proteinuria is a significant pathological feature of feline CKD, resulting primarily from glomerular hypertension and impaired permselectivity. While the predominant histological lesion in cats is tubulointerstitial, the presence of proteins in the urine has substantial clinical relevance. Filtered proteins, including albumin, immunoglobulin G, and transferrin, trigger pro-inflammatory and profibrotic responses upon reaching the tubular lumen. Tubular epithelial cells respond to this protein load by releasing mediators that promote apoptosis, atrophy, and interstitial fibrosis [4,29]. Additionally, the accumulation of non-esterified fatty acids that dissociate from albumin under intratubular pH conditions can exert direct cytotoxic effects on proximal tubular cells.

In feline medicine, the magnitude of proteinuria is generally modest compared to other species, with median urine protein-to-creatinine ratios (UPC) typically ranging from 0.15 in IRIS stage 2 to 0.65 in stage 4 [14]. Nevertheless, persistent proteinuria is a strong predictor of disease progression and is associated with the development of azotemia and reduced survival times, particularly in hypertensive cats [14,16,34].

Parallel to these proteinuric changes, the progressive decline in GFR triggers profound disturbances in mineral metabolism, culminating in renal secondary hyperparathyroidism (RHPTH). This condition arises from a maladaptive cycle involving phosphate retention, reduced calcitriol synthesis, and hormonal dysregulation. As renal phosphorus excretion declines, hyperphosphatemia develops and becomes increasingly prevalent in advanced IRIS stages, significantly accelerating renal fibrosis and soft tissue mineralization [3,35].

In the early stages of CKD, serum phosphorus concentrations often remain within reference intervals due to compensatory mechanisms mediated by fibroblast growth factor-23 (FGF-23) and parathyroid hormone (PTH). FGF-23 correlates inversely with glomerular filtration rate and promotes phosphaturia while suppressing calcitriol synthesis [16]. The resulting calcitriol deficiency removes inhibitory feedback on PTH secretion, leading to sustained parathyroid hormone elevation even in the absence of evident hyperphosphatemia [29].

This endocrine imbalance is further exacerbated by reduced activity of renal 1α-hydroxylase enzyme, impairing the conversion of calcidiol to calcitriol. The combined effects of low calcitriol, hypocalcemia, and phosphate retention stimulate parathyroid gland hyperplasia and persistent PTH secretion. Sustained RHPTH has been associated with increased mortality as it promotes vascular calcification, oxidative stress, and systemic inflammation [3,14]. Evidence shows that dietary phosphate restriction can mitigate these lesions by reducing serum phosphorus, PTH, and FGF-23 concentrations, highlighting the importance of early and sustained control of the mineral axis to slow CKD progression [20].

#### 1.2.4. Anemia, Azotemia, and Uremic Syndrome

As CKD progresses, the cumulative loss of functioning nephrons leads to impairment of the kidney’s excretory and endocrine functions, culminating in anemia, azotemia, and the systemic manifestations of uremic syndrome.

Anemia is a common and clinically relevant complication, affecting approximately 30% to 65% of cats as the disease advances [16,30,36,37]. This condition is typically characterized as normocytic, normochromic, and poorly regenerative [16,36,37]. Its development is primarily attributed to insufficient erythropoietin (EPO) production by the peritubular fibroblast type-1 interstitial cells in the renal cortex [16,37].

Beyond EPO deficiency, additional contributory mechanisms include malnutrition, myelofibrosis, metabolic disturbances, and the accumulation of uremic toxins. Iron deficiency, particularly functional iron deficiency, plays a significant role and is often driven by increased hepcidin expression, an acute-phase protein that limits iron availability during the chronic inflammatory state characteristic of CKD [16,37,38]. Furthermore, gastrointestinal blood loss may exacerbate iron depletion and further worsen anemia [38]. The clinical impact of anemia is substantial, as reduced oxygen delivery to remaining functional nephrons, particularly within metabolically active and hypoxic regions, accelerates renal injury [4,36]. In severe cases, anemia has been identified as a predictor of poor prognosis, with reported median survival times of approximately 100 days [38].

Azotemia is defined by the accumulation of nitrogenous waste products, including creatinine and urea, in the bloodstream as a consequence of reduced GFR. The severity of azotemia correlates strongly with survival outcomes in cats with CKD [39]. When accompanied by clinical signs and systemic biochemical derangements, azotemia progresses to uremic syndrome [40]. This condition reflects advanced renal dysfunction and is characterized by fluid overload, electrolyte imbalances, and multisystemic effects. Urea itself exerts direct and indirect toxic effects, particularly on the nervous system [41].

In cats, specific gut-derived toxins, such as indoxyl sulfate, p-cresyl sulfate, and trimethylamine-N-oxide (TMAO), accumulate as renal function deteriorates. These compounds originate from bacterial metabolism of dietary precursors within the intestine, where the typically high-protein feline diet may play a substantial role in toxin production [42]. Indoxyl sulfate, in particular, promotes tubular inflammation and has been implicated in the progression of renal injury. Progressive retention of these toxins contributes to clinical manifestations such as nausea, vomiting, anorexia, lethargy, and altered mentation, ultimately leading to reduced quality of life and increased morbidity and mortality [41].

Collectively, the structural, hemodynamic, metabolic, and endocrine alterations described above illustrate that feline CKD is a dynamic and multifactorial process that evolves long before evident clinical signs or azotemia become apparent. Progressive nephron loss, fibrotic remodeling, mineral imbalance, and systemic consequences such as anemia and uremia develop in parallel, often in a clinically silent manner. This biological complexity underlies one of the major challenges in feline CKD management: the limited ability of conventional diagnostic tools to detect disease in its early and potentially more modifiable stages. In this context, understanding the strengths and limitations of current diagnostic approaches is essential to bridge the gap between pathophysiological progression and timely clinical intervention.

## 2. Search Strategy

To ensure a comprehensive and objective synthesis of feline CKD progression and biomarkers, a structured literature search was conducted across PubMed, Science Direct, and Google Scholar databases. The search focused on peer-reviewed articles, clinical trials, and consensus guidelines published between 2000 and 2025. Search terms included “feline chronic kidney disease”, “renal pathophysiology”, “tubulointerstitial fibrosis”, “biomarkers”, “SDMA”, “FGF-23”, and “early diagnosis”. Inclusion criteria prioritized longitudinal studies, prospective clinical trials, and the IRIS guidelines. Exclusion criteria were applied to studies with no peer review process, conference abstracts with insufficient data, and studies focusing exclusively on acute kidney injury without relevance to chronic progression. A total of 84 references were selected based on their methodological rigor and clinical relevance to provide a multi-parametric view of the disease.

## 3. Current Diagnostic Landscape

Feline CKD is typically diagnosed based on compatible clinical signs and the presence of renal azotemia, with urinalysis, particularly urine-specific gravity (USG), being essential for confirmation. While advanced CKD is usually easy to identify through blood and urine testing, these approaches have limited sensitivity for detecting early or non-azotemic disease [43].

Since CKD is more common in older cats, close longitudinal monitoring of their health becomes increasingly important with advancing age. Beginning at approximately seven years of age, it is recommended that cats be examined twice a year, including assessment of body weight, body condition, blood pressure, complete blood count, serum biochemistry, and routine urinalysis [20]. If routine findings are inconclusive but clinical suspicion persists, additional diagnostic procedures, such as imaging or histopathology, may assist in identifying underlying structural changes [3].

### 3.1. Medical History, Physical Assessment and Clinical Signs

The clinical presentation of CKD in cats varies greatly, reflecting different stages of the disease’s progression. While some animals are diagnosed incidentally during routine screening, others show only mild signs, or present at terminal stages characterized by emaciation and severe dehydration. Although CKD is typically chronic, sudden clinical worsening is not uncommon. This possibility requires a high index of suspicion, particularly in senior and geriatric cats, although CKD can affect cats of any age [43].

Clinical signs usually appear in more advanced stages, making regular screening essential for high-risk populations [16]. In early stages, CKD rarely causes clinical signs, especially before the onset of azotemia, although progressive weight loss may represent an early indicator [44]. The most common clinical signs are generally nonspecific and include inappetence, polyuria, polydipsia, weight loss, lethargy, halitosis, and vomiting [43,44]. Gastrointestinal manifestations such as anorexia, nausea, melena, and diarrhea are among the most frequent reasons for veterinary consultation, often accompanied by other signs such as muscle wasting, weakness, and urinary incontinence [44].

On physical examination, affected cats may present with a thin body condition, dehydration, periodontal disease, and an unkempt coat. Abnormal renal palpation, revealing small, irregular, or occasionally enlarged kidneys, is a hallmark finding. Additionally, pale mucous membranes may be observed, particularly in cases of non-regenerative anemia [43,45]. A thorough clinical assessment must also account for complications that influence disease progression, such as systemic hypertension, secondary renal hyperparathyroidism, hypokalemia, and metabolic acidosis [16].

A standard diagnostic visit should integrate a detailed history and medication review with a complete physical examination, body weight assessment, and nutritional scoring. This initial clinical assessment provides the necessary context for interpreting laboratory findings, including hematocrit, serum biochemistry, and urinalysis [3].

### 3.2. IRIS Staging and Substaging

The laboratory diagnosis of CKD primarily relies on identifying persistent azotemia combined with reduced urine specific gravity (USG < 1.035), typically assessed through serum biochemistry and urinalysis. USG provides valuable insight into the kidneys’ concentrating ability and, when persistently low, particularly alongside azotemia, supports the diagnosis of renal dysfunction. In these cases, it is important to exclude pre-renal and post-renal causes by evaluating urine concentration, assessing response to fluid therapy, and considering the cat’s clinical history and physical examination [7,20,46].

The GFR is considered the most reliable indicator of kidney function. While its direct measurement represents the most accurate method, it is impractical for routine use in companion animals; therefore, GFR is usually estimated using biomarkers such as urea and creatinine, which only begin to rise after approximately 75% of kidney function has been lost. Creatinine is generally preferred, but its nonlinear relationship with GFR means that small early declines in kidney function cause minimal creatinine increases, whereas large rises later may reflect relatively modest decreases in GFR [46]. More recently, symmetric dimethylarginine (SDMA) has emerged as a more sensitive biomarker as it increases earlier than creatinine and is not influenced by muscle mass, allowing earlier detection of CKD, including in non-azotemic cats. SDMA is now included in the IRIS staging guidelines, improving diagnostic accuracy and facilitating timely intervention [46,47].

CKD staging is implemented once the diagnosis has been confirmed, helping guide treatment decisions and clinical monitoring. When performed early, it enables appropriate therapeutic interventions to be initiated, improving patient outcome [43,48]. As the disease progresses, higher stages are generally associated with shorter survival times, highlighting the importance of accurate and consistent staging [49]. IRIS recommendations suggest that staging be based primarily on fasting blood creatinine and SDMA concentrations, ideally both, measured at least twice in a stable, well-hydrated patient. Following staging, cats are further classified into substages based on the assessment of proteinuria and systemic blood pressure.

Stage 1 CKD in cats is characterized by normal blood creatinine concentrations or normal to mildly increased SDMA levels, alongside other indicators of renal disease, such as impaired urinary concentrating ability, abnormal renal palpation or imaging findings, proteinuria of renal origin, or abnormal biopsy results. Stage 2 involves normal to mildly increased creatinine concentrations with mild renal azotemia and mildly elevated SDMA, often accompanied by minimal or absent clinical signs. Stage 3 is characterized by moderate renal azotemia and may be associated with variable extrarenal clinical manifestations, whereas stage 4 reflects severe renal impairment and is associated with a higher risk of systemic uremic crises [48]. This staging system is intended to be applied during periods of short-term clinical stability, and re-evaluation approximately every six months can assist in monitoring disease progression [50]. Nevertheless, maintaining recommended monitoring frequencies remains a challenge, as owners’ constraints often prevent clinicians from reassessing patients as frequently as guidelines suggest [51]. This highlights the need for better veterinarian–caregiver communication to improve adherence and ensure that an early diagnosis helps minimize the caregiver burden [52].

After staging, CKD is further classified through substaging based on the urine protein-to-creatinine (UPC) ratio and systolic blood pressure (SBP) [50]. Ideally, the UPC ratio should be determined once urinary tract inflammation/infection or hemorrhage has been excluded, using species-appropriate methods, and should be measured in all cats with CKD. Substaging should be based on at least two urine samples collected over a two-week period. Cats with persistent borderline proteinuria should be re-evaluated within two months, as sustained proteinuria is strongly associated with disease progression. In advanced stages of CKD (stages 3 and 4), proteinuria may paradoxically decrease as renal function deteriorates [48].

Arterial hypertension is a common complication of CKD in cats and is associated with a poorer renal prognosis [3]. IRIS recommends that blood pressure be measured in all cats with CKD, using multiple readings obtained in a calm environment to ensure accuracy. Final risk classification is based on several SBP measurements, preferably collected during repeat clinic visits. SBP is used to sub-classify cats according to their risk of target organ damage and the presence of associated complications. When target organ damage is not initially evident, confirming persistent hypertension through repeated measurements over one to two weeks is essential [48] (Table 1).

### 3.3. Traditional Laboratory Markers and Their Limitations

The clinical diagnosis and monitoring of feline CKD have traditionally relied on a combination of hematology, serum biochemistry and urinalysis. While these tools are fundamental in clinical practice, they possess inherent limitations, particularly with regard to early detection and susceptibility to extra-renal influences. Anemia is a common finding, making a complete blood count (CBC) essential to assess hemoglobin and total proteins. Although examination of blood smears and parameters such as mean corpuscular volume (MCV), red cell distribution width (RDW), and mean corpuscular hemoglobin concentration (MCHC) assist in differentiating anemia types, these often remain within reference ranges in CKD-associated anemia. Since this anemia is primarily driven by reduced EPO production, reticulocyte counts typically remain low, and these changes often manifest only when the disease is already established.

Regarding renal function assessment, the GFR is the most sensitive indicator, especially useful in early stages when routine biomarkers are still within normal ranges. While inulin clearance remains the gold standard, its clinical impracticality has led to the use of plasma clearance methods, such as iohexol. Iohexol is a safe, non-metabolized marker excreted almost exclusively by glomerular filtration, allowing for the identification of early reductions in renal function [53,54]. The precision of GFR estimates is fundamentally dependent on the mathematical modeling applied. Bicompartmental analysis is considered the most appropriate pharmacokinetic model as it accounts for the initial distribution phase of the marker between plasma and the extracellular fluid volume (ECFV). In contrast, monocompartmental models assume immediate mixing and distribution, which leads to an underestimation of the area under the curve (AUC) and a subsequent overestimation of GFR. This overestimation is particularly pronounced in uremic patients, where altered elimination kinetics and distribution volumes can shift the time required for the marker to reach equilibrium. Therefore, to balance clinical practicality with diagnostic accuracy, the use of a slope-intercept method with a validated correction formula, such as the one developed specifically for cats (1.036 × GFR uncorrected − 0.062 × GFR uncorrected^2^), is recommended to align monocompartmental results with the more rigorous bicompartmental gold standard. However, despite its reliability, GFR measurement is often bypassed in routine practice due to the requirement for multiple blood samplings [54].

In routine biochemistry, serum creatinine is the most widely used marker and a central component of the IRIS staging system [55,56]. It serves as a surrogate for GFR; however, the relationship between the two is exponential, leading to a significant diagnostic gap whereby creatinine typically rises only after approximately 75% of nephron loss has occurred [57]. This marker is also heavily influenced by muscle mass, meaning that sarcopenic geriatric cats may maintain deceptively low creatinine levels despite severe renal impairment, while certain breeds, such as the Burmese, may exhibit naturally higher baseline levels [55]. Age further contributes to variability, as kittens up to 8 weeks old can present creatinine concentrations similar to or higher than those of adults [58]. Similarly, blood urea nitrogen (BUN) is considered an insensitive and non-specific marker for early CKD, as it is readily influenced by high-protein diets or gastrointestinal bleeding [56].

The emergence of SDMA has addressed some of these limitations. Produced by all nucleated cells and excreted primarily by the kidneys, SDMA exhibits a more linear relationship with GFR and is not affected by muscle mass, potentially revealing reductions in GFR of up to approximately 40% [47,59,60]. While its inclusion in IRIS guidelines has improved early detection, its specificity remains under investigation, and potential breed-related variations require further study [59].

Progression of CKD also triggers profound disruptions in mineral metabolism. Phosphorus retention drives renal secondary hyperparathyroidism and soft tissue mineralization, yet serum phosphorus concentrations often remain within reference ranges in IRIS stages 1 and 2 due to compensatory mechanisms mediated by FGF-23 and PTH [3,55,61]. Calcium assessment is equally complex and represents a critical component of chronic kidney disease–mineral and bone disorder (CKD-MBD), a systemic syndrome involving mineral, bone, and cardiovascular calcification abnormalities. Ionized calcium (iCa) is the diagnostic gold standard, as total calcium (tCa) is notoriously unreliable in feline CKD due to its susceptibility to hypoalbuminemia and acid–base disturbances, which alter calcium–protein binding. Despite a high specificity of 92%, tCa has only a 30% sensitivity for identifying hypercalcemia in CKD cats. Clinical studies indicate that while normocalcemia is frequent, the prevalence of ionized hypercalcemia can reach up to 30%, whereas ionized hypocalcemia is more often associated with advanced stages and severe secondary hyperparathyroidism [62,63].

Electrolyte disturbances such as hypokalemia further complicate clinical interpretation, as serum potassium concentrations typically transition from a 20-30% prevalence of hypokalemia in IRIS stages 2 and 3 to hyperkalemia in terminal stages due to severe GFR decline [3,64]. This variability, coupled with the fact that blood potassium may not accurately reflect total body stores, as approximately 95% of potassium is intracellular, limits its reliability as a marker of disease stability [55]. Similarly, biochemical markers of acid–base balance, such as bicarbonate and total CO_2_, are often insensitive for early detection, as compensatory mechanisms typically mask metabolic disturbances until advanced stages. However, metabolic acidosis is a key driver of CKD progression. In cats, impaired organic acid excretion leads to titration acidosis with an increased anion gap. This state promotes protein catabolism, muscle wasting, and bone resorption, while also accelerating tubulointerstitial fibrosis. Furthermore, acidosis triggers an extracellular potassium shift, which may mask total body depletion [65].

Additionally, alterations in lipid markers are frequently overlooked in routine panels, despite histological evidence of renal lipid accumulation in up to 89% of cats with CKD. This high prevalence suggests that dyslipidemia may contribute to early metabolic disturbances that traditional laboratory markers fail to capture while appearing clinically stable [23,66].

Urinalysis remains a central diagnostic pillar; however, its components often fail to reflect the early stages of disease progression [67]. While a USG ≤ 1.035 in conjunction with persistent azotemia is a classic diagnostic criterion, this parameter is easily influenced by non-renal factors such as diet and water intake, and may remain relatively preserved in some cats despite significant parenchymal loss [54,68]. Furthermore, sediment analysis is essential to distinguish renal proteinuria from inflammatory causes, but its reliability is hindered by rapid cellular degeneration and temperature-induced artifacts [69]. Similarly, urine cultures often reveal subclinical bacteriuria that does not correlate with creatinine levels or disease progression, complicating this distinction between clinically relevant infection and incidental findings [70,71,72].

Finally, assessment of proteinuria is crucial for predicting survival, yet current methods present significant technical limitations. Urine dipstick testing is notoriously unreliable in cats, with a specificity of only 31%, largely due to false-positive results associated with alkaline urine or active sediment. Although the sulfosalicylic acid (SSA) test detects a broader range of proteins, its specificity is even lower, at 25%, and reliance on visual turbidity grading renders it highly subjective [73,74]. The UPC ratio is the standard method for quantification, and even mild elevations (≈0.4) are associated with reduced survival. However, UPC testing fails to detect albumin concentrations below the standard detection threshold of 30 mg/dL, creating a diagnostic gap that can only be addressed through microalbuminuria (MA) testing. MA assays detect albumin concentrations as low as 1 mg/dL, offering a substantially more sensitive tool for identifying cats at risk of early renal injury [73,75].

## 4. Emerging Biomarkers: Bridging the Diagnostic Gap

The search for novel biomarkers is driven by the need to identify renal compromise before irreversible nephron loss occurs. FGF-23 has emerged as a key indicator of early metabolic derangement, as its plasma concentrations rise progressively alongside declining kidney function. It effectively serves as an early warning for phosphate retention; indeed, azotemic cats with hyperphosphatemia exhibit significantly higher FGF-23 levels than normophosphatemic cats at the same disease stage. This observation suggests that FGF-23 may reveal disturbances in mineral metabolism while serum phosphorus concentrations still appear deceptively stable. Nevertheless, reference intervals in geriatric cats are broader than those in humans, potentially reflecting species-specific adaptations or dietary influences that necessitate careful clinical interpretation [76].

Serum cystatin C (sCysC) is also being explored as a GFR marker that remains unaffected by muscle mass, addressing a major flaw of creatinine. Although sCysC is widely used in humans and moderately in canine medicine, its use in cats is still limited by the lack of species-specific assays. Current methods often fail to distinguish clearly between healthy cats and those with early CKD, leading to overlapping results. Furthermore, common comorbidities such as hyperthyroidism can interfere with sCysC concentrations, reducing its reliability as a standalone diagnostic marker [59].

Shifting the focus toward structural renal integrity, urinary biomarkers of tubular injury provide a more granular assessment than traditional filtration-based markers. Urinary cystatin B (uCysB) and retinol binding protein (RBP) are particularly promising indicators of ongoing tubular injury. Notably, uCysB can detect active tubular damage even when serum creatinine remains stable, effectively narrowing the diagnostic gap for monitoring disease progression [77,78,79]. Other markers, such as N-acetyl-β-D-glucosaminidase (NAG) and neutrophil gelatinase-associated lipocalin (NGAL), also show a strong correlation with tubular injury. However, their clinical utility remains inconsistent due to high interindividual variability and confounding factors, including urinary tract inflammation and hyperthyroidism, which may artificially elevate biomarker levels and complicate differentiation between primary renal damage and transient systemic stress [59,80].

Table 2 provides a comprehensive overview of feline CKD biomarkers, showing that while emerging tools are essential for bridging the diagnostic gap, they also present specific limitations that necessitate integration with conventional laboratory markers rather than isolated interpretation. Emerging biomarkers therefore complement, rather than replace, traditional diagnostic parameters by enabling earlier detection of renal compromise before irreversible nephron loss occurs.

### Clinical Hierarchy and Practical Application

To assist practitioners in navigating the diagnostic weight of these tools, a clear distinction must be made between routine practice and research-grade markers. While established biomarkers such as SDMA are now integrated into many reference laboratory panels, several emerging proteins, particularly those indicating acute tubular insult, remain primarily within the scope of academic research. Table 3 provides a practical hierarchy of these biomarkers, categorizing them by their current clinical availability and utility in a practice setting.

## 5. Clinical Monitoring and Prognostic Value

Regular assessments are fundamental to managing the progressive nature of feline CKD, as the clinical needs of these patients often shift over time. The frequency of evaluation depends largely on the IRIS stage and clinical stability. Cats in stages 1 and 2 typically require monitoring every 4 to 6 months, whereas those in stages 3 and 4 usually necessitate more frequent visits, approximately every 3 to 4 months. In cases of rapid progression, persistent proteinuria, or uncontrolled hypertension, evaluations may be required as often as every 1 to 4 weeks during stabilization phases [3,20].

A standard monitoring visit must integrate a thorough medical history with a complete physical examination, with particular attention to body weight and nutritional status. Serial measurements of BUN, creatinine, and phosphorus are essential for tracking the rate of functional decline. Furthermore, assessment of the mineral axis should rely on iCa and PTH to ensure adequate control of renal secondary hyperparathyroidism, particularly in cats receiving calcitriol therapy. Given that tCa frequently fails to reflect the true ionized status in feline CKD, direct measurement of iCa is essential for accurate clinical decision-making [29,62]. In this context, emerging biomarkers such as FGF-23 may provide additional insight into treatment efficacy and early metabolic disturbances that precede overt hyperphosphatemia [29]. Adjunctive nutritional strategies may also contribute to CKD management, particularly dietary supplementation with omega-3 polyunsaturated fatty acids (EPA/DHA). A recent systematic review in companion animals with neoplastic and non-neoplastic diseases supports consistent anti-inflammatory and lipid-modulating effects of EPA/DHA supplementation, which may be biologically relevant to chronic kidney disease progression [81].

The prognosis for cats with CKD is highly individual and often difficult to predict, as many patients remain stable for years while others experience a more rapid decline [82]. Several modifiable risk factors are directly linked to reduced survival, including poor body condition, anemia, and hyperphosphatemia. Notably, persistent proteinuria and systemic hypertension remain two of the strongest predictors of a poor outcome. Despite these risks, the progression of feline CKD is typically gradual. Veterinary nurses and technicians play a crucial role in long-term disease monitoring, supporting owners and identifying subtle changes in parameters such as body condition or the UPC ratio that should prompt a more detailed clinical evaluation [3,20,82].

### The Clinical Paradox of Early Diagnosis and the Ethics of Analgesia

A critical challenge in the clinical application of early biomarkers is the “utility paradox”: the concern that identifying CKD in its non-azotemic stages may lead to negative therapeutic interventions. A primary risk is the inappropriate withdrawal of necessary medications, particularly NSAIDs for managing comorbidities such as osteoarthritis. Chronic pain has a profound negative impact on feline quality of life and impairs the owner–cat bond, yet its treatment is frequently neglected due to renal concerns [83].

Evidence from landmark studies indicates that the use of NSAIDs in stable, well-monitored CKD cats does not necessarily accelerate the decline of renal function. Specifically, long-term maintenance therapy with meloxicam (at a median dose of 0.02 mg/kg/day) has been demonstrated to be safe for aged cats with stable CKD and may even slow disease progression in some individuals [84]. Therefore, the identification of early renal compromise should be viewed as a mandate for safer, proactive management and monitoring of treatment tolerance, rather than a reason to withhold necessary analgesia. This approach ensures a holistic clinical balance where the management of chronic pain is refined by earlier diagnosis, rather than restricted by it.

## 6. Conclusions and Future Directions

The management of feline CKD is currently undergoing a transformative shift by moving from reactive stabilization toward a more proactive and personalized approach. The translational potential of novel biomarkers such as uCysB, NGAL, and FGF-23 lies in their ability to detect subclinical renal distress before the threshold of irreversible nephron loss is crossed. Future directions point toward the integration of multi-parametric diagnostic panels that combine filtration markers with indicators of active tubular injury and metabolic derangement. This paradigm shift aims to redefine the diagnostic gap by enabling clinical intervention during the early stages of fibrosis and mineral imbalance, when therapeutic strategies are most likely to slow disease progression effectively. Nonetheless, it is essential to recognize that these emerging markers are not infallible; their clinical utility is still often constrained by biological variability, interference from common comorbidities such as hyperthyroidism, and a lack of standardized species-specific reference intervals. These limitations underscore the need for cautious interpretation and integration alongside established diagnostic parameters.

However, the path toward widespread clinical adoption faces significant practical challenges. High costs and the limited availability of species-specific assays mean that many of these emerging biomarkers remain restricted to research or referral settings. Until these barriers are addressed, traditional diagnostic tools remain indispensable. Urinalysis, in particular, continues to be a cornerstone of feline practice, as its accessibility and high negative predictive value make it a critical tool for excluding complications and monitoring clinical stability. This remains especially relevant in real-world clinical settings where financial constraints restrict access to advanced diagnostic technologies.

In conclusion, understanding the progression of feline CKD requires a holistic view that bridges the gap between molecular pathophysiology and clinical reality. While serum creatinine and SDMA are the current pillars of staging, they represent only static snapshots within a dynamic and evolving pathological process. However, this diagnostic progress must be balanced with clinical reality. Early detection should not lead to unnecessary medical intervention, but rather to a personalized approach that prioritizes the cat’s comfort. In geriatric patients, renal preservation must go hand in hand with pain management and the reduction in clinic-related stress. Ultimately, the value of these new biomarkers lies not just in improving laboratory numbers, but in ensuring good quality of life and supporting the bond between the owner and the cat throughout the disease. The integration of emerging biomarkers with consistent clinical monitoring focused on body condition, mineral homeostasis, and preservation of remaining renal function offers the most promising strategy for extending both survival and quality of life in aging cats. Ultimately, the goal is to shift feline CKD from a disease primarily managed at advanced stages to one that can be anticipated and intercepted earlier, promoting healthier aging in the feline population.

## Figures and Tables

**Table 1:** IRIS staging and substaging system for feline CKD.

Classification Parameter	Reference Interval	Stage/Substage **	Clinical Significance
I. Primary staging			
Blood creatinine μmol/L mg/dL	<140 <1.6	Stage 1	Non-azotemic; presence of renal abnormalities
	140–250 1.6–2.8	Stage 2	Mild renal azotemia; clinical signs absent or present
	251–440 2.9–5.0	Stage 3	Moderate renal azotemia; systemic clinical signs may be present
	>440 >5.0	Stage 4	Severe renal azotemia; increased risk of uremic syndrome
SDMA μg/dL	<18	Stage 1	Non-azotemic; presence of renal abnormalities
	18–25	Stage 2	Mild renal azotemia; clinical signs absent or present
	26–38	Stage 3	Moderate renal azotemia; systemic clinical signs may be present
	>38	Stage 4	Severe renal azotemia; increased risk of uremic syndrome
II. Substaging based on proteinuria			
UPC value	<0.2	Non-proteinuric	Potential microalbuminuria
	0.2–0.4	Borderline proteinuric	Requires monitoring; potential microalbuminuria
	>0.4	Proteinuric	Strong predictor of disease progression
II. Substaging based on blood pressure			
Systolic blood pressure mmHg	<140	Normotensive	Minimal risk of future lesions in target organs
	140–159	Prehypertensive	Low risk of future lesions in target organs
	160–179	Hypertensive	Moderate risk of future lesions in target organs
	>180	Severely hypertensive	High risk of future lesions in target organs

** Staging is primarily based on fasting blood creatinine and/or symmetric dimethylarginine (SDMA) concentrations. Substaging is subsequently performed according to proteinuria status (urine protein-to-creatinine ratio, UPC) and systolic blood pressure (SBP), allowing standardized clinical classification and assessment of risk for target organ damage. Adapted from International Renal Interest Society (IRIS) guidelines (2023) [48].

**Table 2:** Overview of established and emerging biomarkers for feline CKD.

Marker	Sample	Primary Application	Clinical Limitations /Challenges	Availability	Reference
Gold Standard					
Iohexol	Serum	Precise GFR measurement	Clinically impractical; requires multiple blood samplings	Specialized	[53,54]
iCa	Serum	Mineral status	Requires anaerobic handling; essential to bypass tCa unreliability	Commercial	[62,63]
Traditional					
Creatinine	Serum	GFR surrogate	Low sensitivity; affected by muscle mass, breed and age	Commercial	[55,56,57,58]
BUN	Serum	Nitrogenous waste	Easily skewed by high-protein diets or gastrointestinal bleeding	Commercial	[56]
Phosphorus	Serum	Mineral metabolism	Often masked by FGF-23/PTH compensation in early stages	Commercial	[3,55,64]
Potassium	Serum	Electrolyte stability	Serum levels do not reflect total body stores	Commercial	[3,55,64]
tCa	Serum	Mineral balance	High specificity but very low sensitivity	Commercial	[62,63]
USG	Urine	Renal concentrating function	Influenced by non-renal factors; may remain normal in early CKD	Commercial	[54,68]
UPC ratio	Urine	Proteinuria/substaging	High variability; requires exclusion of inflammation/UTIs	Commercial	[73,74,75]
Established					
SDMA	Serum	Early/stable GFR assessment	Specificity under investigation; potential breed-related variations	Commercial	[47,59,60]
Emerging					
FGF-23	Plasma/serum	Early indicator of mineral derangement	Broad reference intervals in geriatric cats; requires careful interpretation	Specialized	[76]
sCysC	Serum	Muscle-independent GFR marker	Lacks species-specific tests; overlapping results; confounded by hyperthyroidism	Research only	[59]
uCysB	Urine	Active tubular injury detection	Detects damage while creatinine remains stable; limited clinical availability	Emerging	[77,78,79]
RBP	Urine	Proximal tubule damage marker	Primarily used in research settings; specific for reabsorption failure	Research only	[77,78,79]
NGAL/NAG	Urine	Correlation with tubular insult	High individual variability; skewed by UTIs or systemic stress	Research only	[59,80]

Table summarizes the clinical utility and main limitations of currently available biomarkers used in feline CKD. Direct GFR measurement using iohexol clearance remains the reference method but is rarely applied in routine clinical practice. Traditional markers are widely used for staging but may be influenced by extra-renal factors. Established biomarkers such as symmetric dimethylarginine (SDMA), together with emerging markers including fibroblast growth factor 23 (FGF-23) and urinary cystatin B (uCysB), may improve early detection of metabolic and tubular alterations before advanced nephron loss. Availability is categorized as: Commercial (routine veterinary laboratories); Specialized (reference laboratories or specialized equipment); Emerging (recently validated/limited market availability); and Research only (currently restricted to investigative settings). BUN, Blood Urea Nitrogen; iCa, Ionized Calcium; NAG, N-acetyl-β-D-glucosaminidase; NGAL, Neutrophil Gelatinase-Associated Lipocalin; PTH, Parathyroid Hormone; RBP, Retinol-Binding Protein; sCysC, Serum Cystatin C; tCa, Total Calcium; UPC, Urine Protein-to-Creatinine ratio; USG, Urine-Specific Gravity; UTI, Urinary Tract Infection.

**Table 3:** Clinical applicability of emerging biomarkers.

Marker	Clinical Status	Practitioner’s Takeaway	Reference
SDMA	Established	Use for early screening; commercially available in most reference laboratories	[47,59,60]
FGF-23	Available	Useful for monitoring mineral disorders; requires specialized referral laboratories	[76]
sCysC	Low Utility	Not recommended for routine use due to lack of feline-specific validation and thyroid interference	[59]
uCysB	Emerging	High potential for detecting active tubular injury, but limited commercial access	[77,78,79]
RBP	Research	Restricted to academic settings for proximal tubule assessment; reflects reabsorption failure	[77,78,79]
NAG/NGAL	Research	Valuable for scientific studies; not currently recommended for routine clinical use	[59,80]

This table categorizes biomarkers based on their current clinical availability and diagnostic weight to assist practitioners in differentiating between validated tools and research-grade markers. FGF-23, Fibroblast Growth Factor 23; NAG, N-acetyl-β-D-glucosaminidase; NGAL, Neutrophil Gelatinase-Associated Lipocalin; RBP, Retinol-Binding Protein; sCysC, Serum Cystatin C; SDMA, Symmetric Dimethylarginine; uCysB, Urinary Cystatin B.

## Data Availability

Not applicable.
